# Deep learning for behaviour classification in a preclinical brain injury model

**DOI:** 10.1371/journal.pone.0268962

**Published:** 2022-06-15

**Authors:** Lucas Teoh, Achintha Avin Ihalage, Srooley Harp, Zahra F. Al-Khateeb, Adina T. Michael-Titus, Jordi L. Tremoleda, Yang Hao

**Affiliations:** 1 School of Electronic Engineering and Computer Science, Queen Mary University of London, Mile End, London, United Kingdom; 2 Centre for Neuroscience, Surgery and Trauma, The Blizard Institute, Barts and The London School of Medicine and Dentistry, Queen Mary University of London, London, United Kingdom; Universitatsklinikum Wurzburg, GERMANY

## Abstract

The early detection of traumatic brain injuries can directly impact the prognosis and survival of patients. Preceding attempts to automate the detection and the assessment of the severity of traumatic brain injury continue to be based on clinical diagnostic methods, with limited tools for disease outcomes in large populations. Despite advances in machine and deep learning tools, current approaches still use simple trends of statistical analysis which lack generality. The effectiveness of deep learning to extract information from large subsets of data can be further emphasised through the use of more elaborate architectures. We therefore explore the use of a multiple input, convolutional neural network and long short-term memory (LSTM) integrated architecture in the context of traumatic injury detection through predicting the presence of brain injury in a murine preclinical model dataset. We investigated the effectiveness and validity of traumatic brain injury detection in the proposed model against various other machine learning algorithms such as the support vector machine, the random forest classifier and the feedforward neural network. Our dataset was acquired using a home cage automated (HCA) system to assess the individual behaviour of mice with traumatic brain injury or non-central nervous system (non-CNS) injured controls, whilst housed in their cages. Their distance travelled, body temperature, separation from other mice and movement were recorded every 15 minutes, for 72 hours weekly, for 5 weeks following intervention. The HCA behavioural data was used to train a deep learning model, which then predicts if the animals were subjected to a brain injury or just a sham intervention without brain damage. We also explored and evaluated different ways to handle the class imbalance present in the uninjured class of our training data. We then evaluated our models with leave-one-out cross validation. Our proposed deep learning model achieved the best performance and showed promise in its capability to detect the presence of brain trauma in mice.

## Introduction

The detection and effective evaluation of traumatic brain injuries (TBIs) remain critical in the treatment and prognosis of these injuries. TBIs involve an initial injury caused by a mechanical insult, which in most severe cases can be fatal [1–3]. Survivors of the initial impact injury often experience subsequently a progressive brain damage with long-term implications [4]. A progressive neuroinflammatory response can persist for years and lead to significant neurodegenerative conditions with cognitive, sensory, and psychiatric dysfunctions [5, 6]. With no effective way to overcome the primary injury, the current approaches to treating traumatic injuries rely on early diagnostic and immediate supportive actions to modulate the progressive neuroinflammatory response to minimise the harm associated with the progressive secondary injury [7].

Therefore, early diagnosis of TBI remains critical, in order to implement immediate supportive treatments. Unfortunately, the diagnostic approaches remain mostly linked to the diagnosis of individual patients, and there are limited prospective diagnostic approaches that could help to identify the presence and prognosis of a said injury in a less subjective manner and based on a larger comprehensive population database. This would be particularly relevant for patients with mild brain injury that show limited clinical signs. Most diagnostic tests such as the Glasgow Coma Scale describe the extent of impaired consciousness based on eye, motor, and verbal responses [8]. Other tests such as the grip strength test [9] typically focus on assessing patients by monitoring how they perform a physical task. Such tests carried out on individual patients may fall short of providing a more comprehensive perspective on the overall activity and physical wellbeing, related to larger population studies. Similar challenges are encountered when preclinically modelling the disease, where objective assessments remain challenging. Such evaluation methods may provide a direct and markedly subjective observation data based on existing latent knowledge and patterns, yet developing new ways to understand a large data population and how this may be able to support an early and more predictive diagnostic information is likely to result in better understanding and prognosis of the disease. Such knowledge would be very valuable to establish earlier diagnostics and supportive treatments [10].

The use of expert systems for TBI analysis can further elevate the reliability when investigating impairments linked to brain injuries and the progressive neurodegenerative changes. Classification implementations that involve machine learning and deep learning are able to study patterns in the physiology of the brain not easily discernible by other means [11]. Importantly, the development and testing of different machine learning methods in preclinical models of TBI may help to establish and validate new diagnostic and prognosis approaches to evaluate disease progression in patients recovering from brain injury, and target better informed supportive treatments and rehabilitation programs.

### Classification of brain injuries

Early attempts to detect TBIs using machine learning featured the use of the support vector machine (SVM) with electroencephalography (EEG) [12]. Cao et al. [12] used EEG signals which monitor electrical activity in the brain, to classify the presence of residual functional abnormalities originating from earlier concussions suffered by athletes.

Present methods that employ machine learning and deep learning in order to access underlying knowledge mostly involve convolutional neural networks (CNNs) which are applied on imaging data [13–16]. Imaging modalities such as computerised tomography (CT) scans allow for a convenient implementation of deep learning through the use of CNNs, which are effective in extracting features from images in order to derive patterns in the dataset [17].

Other approaches such as measurements of the intracranial pressure, blood flow, brain tissue metabolism, oxygenation and electrophysiology have been used to monitor the physiology of the brain following injury and to monitor tissue damage and cellular impairment [18]. While feasible, these approaches may require invasive diagnostic protocols, provide limited details on behaviour impairments and might pose challenges when assessing larger populations.

Recent developments in preclinical animal studies have suggested a more accessible alternative of investigating the impact of brain injuries using non invasive and continuous monitoring of individual behavioural changes in grouped housed animal cohorts whilst in their home cages [19]. Data obtained from such home cage automated (HCA) monitored behaviour provides large and comprehensive activity and physiological individual data from grouped housed animals, overcoming the constraints towards CNNs imposed by imaging modalities and therefore allows for more flexibility in data processing and model architecture.

### Classifying animal behaviour

The correlation between a comprehensive panel of individual patterns of behaviour and the presence of TBI in a modelling study may be able to provide a substantial data set that could be used for machine learning model training to develop new computational diagnostic approaches that may be applied to TBI classification. Several studies have shown the feasibility of using machine learning techniques that are more sophisticated than the SVM to classify an animal’s behaviours for the purpose of evaluating their well-being. Nadimi et al. [20] uses animal movement on artificial neural networks to classify animal behaviour. Data collected on the head movements of a herd of sheep is fed into a simple feedforward neural network, a multilayer perceptron, to classify the activity of sheep (grazing, lying down, walking, standing and others). Sheep that displayed abnormal activity could show a reasonable cause for suspicion for illnesses or even traumatic injury. A similar multi-layer feed forward neural network is also used in Gutierrez et al. [21] to classify the behaviour of horses based on similar sensory information. The addition of their online monitoring system allows for the real-time classification using their pre-trained neural network.

### Classifying emotions

A similar approach taken to classifying behaviour can also be implemented to classify emotions. Dominguez et al. [22] propose a system that classifies emotions (amusement, sadness and neutral) from photoplethysmogram (PPG) and galvanic skin response (GSR) signals. PPG detects blood volume changes whereas GSR measures sweat gland activity. The signals obtained resulted in 27 features, in which stepwise regression was performed to select the best features. Classification methods such as SVMs, linear discriminant analysis, multinomial regression, decision trees and naive Bayes were then used on features that were obtained and selected from the signal data. The implementation of deep learning to classify emotions was proposed by Kanjo et al. [23]. They utilise the EnvBodySens dataset which is composed of 3 groups of data: on-body (heart rate, GSR, temperature, motion data), location data and environmental data (noise levels, UV, air pressure) [24]. They train their models separately on the three aforementioned groups of data, and then on a fourth group which is a combination of all previous groups. The architecture of their deep learning model consists of a combination of CNN and long short-term memory (LSTM) layers, where the final fully connected layer in the CNN module is fed into an LSTM cell.

Khan et al. [25] implement a similar deep learning model architecture where they merge a CNN and LSTM to classify emotions. Instead of the data from EnvBodySense, which makes use of on body sensors, Khan et al. [25] measure heartbeat and breathing signals through the use of radio frequency (RF) signal reflections off the body. Their unique implementation of deep learning allows for the combined learning of the raw and processed RF signals. The raw signal exists in the typical form of a time-sequence data, which undergoes continuous wavelet transformation (CW) in order to obtain the processed signal. The wavelet transformation was developed for the purpose of time-frequency analysis, which contains knowledge where the definite local coordinates of important properties can be found in the signal [26]. The transformation process represents the one-dimensional (1-D) RF signal as a 2-D scaleogram, visualising various features contained in the signals [27]. The resulting representation takes the form of an image which allows for a more visual and hence presentable analysis of the signal. This image can then be used as a secondary input to complement the 1-D raw signal features in their proposed ‘Y’ shaped neural network. The raw signal is processed through two convolutional-1D layers followed by an LSTM layer, similar to that from Kanjo et al. [23]. The CW transformation is concurrently processed by a pair of convolution-2D layers which is then concatenated with processed raw signal. The concatenated signal is then put through a softmax layer which then provides the prediction in the form of a probability distribution.

Here, we propose a similar deep learning model that can learn from both time-series raw data and their CW transformations through an architecture that combines a CNN with a LSTM unit, which is well-suited to process sequential data. The proposed model is used to assess the presence of brain injury using a preclinical model validation approach. Data was obtained from individual mice monitored in their home cages and cohort groups. We identify the status of mice on whether they are injured, have undergone TBI, or if they are uninjured. We evaluate the feasibility of this model by comparing its performance to 3 other shallow machine learning approaches on their ability to detect changes on an independent test set. The benefits of the proposed model could demonstrate the feasibility of expert systems for traumatic injury evaluation to use different sources of data on unconventional deep learning architectures.

## Materials and methods

Through the use of machine learning, most related works were able to perform classification on behaviour, including functional impairments associated to TBI by using signals obtained from sensors. It was however, only the deep learning emotion classifiers that were able to implement sequential knowledge in their classification techniques. Our approach to implementing deep learning for the severity impact of traumatic injury in a validated mouse model of TBI could adopt a similar method to the deep emotion classifiers, where ordered, sequential data is used to train the model. The classification models are designed to classify three injury states; naive, which are not subjected to any injury; injured, in which the mice have been subjected to a controlled cortical injury (TBI); and shams (craniotomy- as controls), where the mice have undergone sham surgical intervention without brain injury.

### Ethical statement

For this study, the dataset analysed was extracted as part of another ongoing study, to avoid the use of extra animals while maximising animal experimental data in accordance with the 3Rs principles for animal research (Replacement, Reduction and Refinement). All procedures were approved by the Queen Mary University of London (QMUL) Animal Welfare and Ethical Review Body, and executed under an approved Project Licence, in compliance with the UK Animals (Scientific Procedures) Act.

### Animal care and husbandry

A total of 16 adult mice (Crl:CD1(ICR); Charles River, UK) were used for this exploratory study which is carried out as a part of another ongoing study. N = 4 as naive, n = 6 for the TBI group and n = 6 for the craniotomy group were used based on the n values published in our previous study [28]. Behaviour data was automatically acquired and analysed. For this initial study, only male mice were used following the existing literature and our group’s expertise on the characterisation of male mice brain injury models, based on higher incidence of TBI in the male population (17% in male vs 9% in female, see ref. [29]). This work should be followed by further studies in female mice in near future. Animals were grouped housed, within the same cohorts from weaning to avoid any fighting and/or aggression.

Animals were housed in independently ventilated cages (IVC; 1300 cm2; Allentown Europe, UK) under controlled conditions 21±1°C, 40–60% relative humidity, 12hr:12hr light: dark cycle (07:00–19:00 light; 19:00–07:00 dark), with standard mouse diet (PicoLab^®^ Mouse Diet 20; www.labdiet.com) and water available ad libitum. Wood chip bedding with shredded nesting paper, two cardboard tunnel and wood sticks were used. Experimental recording were carried out with animals housed groups of n = 3 or 4 animals.

### Injury modelling and behaviour recording

Experimental TBI was modelled using a controlled cortical impact [30]. Briefly, after 1-week of acclimatisation, anaesthetized mice (ketamine: 50 mg/kg and medetomidine: 0.5 mg/kg; i.p.) were subjected to a unilateral 3.5 mm craniotomy and a controlled cortical injury was carried out on the right hemisphere 2.0 mm behind bregma and 2.5 mm lateral to the midline, using a 3 mm impactor tip with a speed of 3 m/s, a depth of 2.2 mm and a dwell time of 100 ms, applied using the PCI3000 Precision Cortical Impactor™ (Hatteras Instruments, Inc., US). The skull flap was placed back unfixed to allow for expansion, and the skin was sutured. Analgesia (buprenorphine 0.05 mg/kg, s.c.) was used in all animals pre-emptive and post-operatively (up to 3 days post-surgery). The sham control group underwent craniotomy only. Naive animals were not subjected to any intervention (and did not receive analgesia). A modified Neurological Severity Score (mNSS) was used to assess the motor ability, alertness and balance of each animal following intervention (see S1 Fig).

The mice were randomly assigned to either an interventional (CCI; n = 6) or craniotomy group (n = 6); n = 4 naive animals were used for a week for baseline recordings. All mice were tagged with an RFID sensor, subcutaneously placed in the lower flank area aseptically at the time of the surgical intervention; or under a short induction of gas anesthesia (isoflurane 3% in 100% *O*_2_) 24 h prior recording for the naive animals.

**Fig 1:**
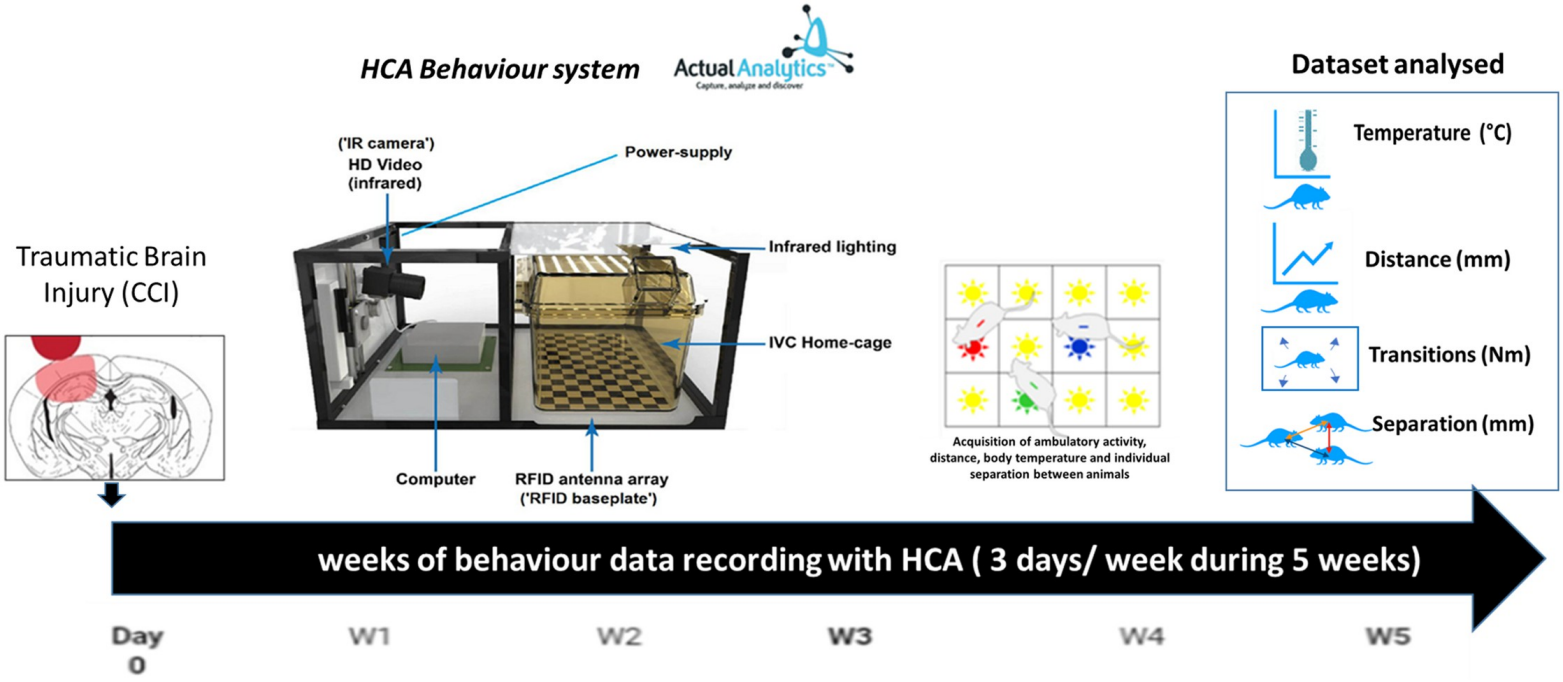
Representative scheme of the experimental behaviour data acquired for the study. Briefly, animals were subjected to a controlled cortical injury via a craniotomy to expose the cortical brain **(CCI- TBI injured group)**, a craniotomy alone without cortical injury **(Sham craniotomy group)**, all under aseptic surgery and anaesthesia with analgesic support. All animals were implanted with a RFID chip. **Naive** animals were implanted with RFID under a brief anaesthesia, with no other interventions. Animals were grouped housed in their home cages, which were placed on top of the baseplate RFID reader and exposed to a side video camera from the Home Cage Analyser (ActualHCA™) system. Non-disturbance recordings were set up as 15 min timeframes and throughout 3 days periods per week, during 5 weeks post-intervention. Naive data was recorded only for a single week as a baseline data. Automated data acquired via the HCA systems included body temperature per individual animal (ºC), distance travelled per individual animal (mm), number of transitions between RFID set up detectors (Nm) and average separation between animals (mm). This time-series raw data was then pre-processed and utilised to train our machine learning models.

Following the surgical intervention, an automated home cage recording system (Home Cage Analyser (ActualHCA™) system, Actual Analytics Ltd, UK) that tracks the RFID signal from each individual mouse was used to record the behaviour [31]. The mice IVC cages are specially fitted directly on top of the baseplate RFID reader, and the system is supported by an infrared lighting panel. The behaviour recordings were set up in consecutive 5 week periods for all groups (with random allocation of the TBI and craniotomy sham control; one week baseline recordings for the naive groups). Fig 1 shows the experimental setup.

### Dataset

Data was recorded in 15 minute intervals, for a period of 72 hours each week for 5 weeks for the TBI and control groups; for one week period for the naive group. RFID Data was then pooled using the Actual HCA Analyser™ software (Actual Analytics Ltd, Edinburgh UK) yielding a csv (comma separated values) file containing raw behavioural data, of multiple variables including the studied parameters below.

**Fig 2:**
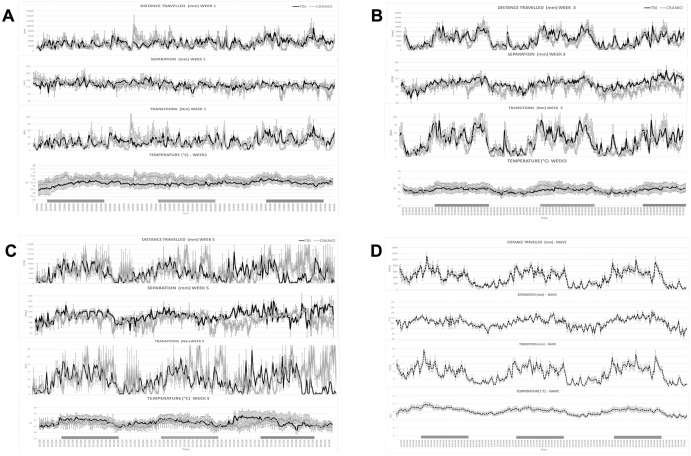
Representative graphs showing the data acquired from the Home Cage Analyser (ActualHCA™) system, for the TBI-Injured, Sham-craniotomy and Naive animals. Parameters analysed include the distance travelled per animal (mm), number of transitions –as spatial transitions between the different RFID sensors, the distance between animals grouped housed in the same cage (mm) and the body temperature between individual animal (ºC). Data is shown as average ±SEM from the grouped animals per cage, plotted from the individual RFID recordings from each animal. Fig 2A–2C show data for the TBI-Injured and the sham-craniotomy groups, recorded during Week 1, Week 3 and Week 5 post-intervention, respectively. Fig 2D shows the recordings acquired for Naive animals, as a one-week baseline reference. Data shown covers a larger study cohort of 35 animals, including n = 16 as naive, n = 11 for the TBI group and n = 8 for the craniotomy group, including the cohorts used deep-learning modelling, as part of a larger ongoing recording project. All raw data used for ML analysis is provided as S1 File.

A representation of the visual data automatically collected from the HCA system is shown in Fig 2. The graphics exemplify the recorded data for W1, W3 and W5 of the study for the CCI and the craniotomy groups, along with a week baseline for naive animals. This data shows the large amount of descriptive data available through recordings, with the CCI and craniotomy control groups showing a poor circadian rhythm during the Week 1 post-injury when compared to naive animals.

### Features

From the data collected by observing the mice, we selected four relevant features, which were then used to train and evaluate our machine learning models. The features used are as follows:

- **Distance travelled**: Total distance that the mouse has travelled in the 15 minute time frame, recorded in millimetres (mm).
- **Temperature**: Body temperature of the mouse across the time of recording, recorded in Celsius (°C).
- **Separation**: Separation, in millimetres (mm), of the observed mouse from other mice.
- **Transition**: The number of times the mouse moves between the different sections of the enclosure, which are sectioned out into grids (12 grids accross a total area of 36×48cm).

### Data pre-processing

With each subject being observed for 72 hours, and data recorded every 15 minutes, we are able to obtain 290 data points for each individual animal for each period of weekly observation. These 290 data points are then split into 6 bins, which stood out when tested amongst 1 and 20 bins, giving the best initial performance for models when tested. Each bin is given either a class of 0 for uninjured (naive), a class of 1 for brain injured (TBI), or a class of 2 for sham controls. The binning results in a dataset size of 708, with 96 naive observations, 324 injured observations and 288 sham observations; presented in Table 1.

**Table 1:** Outline of class distribution in the dataset.

Class	Number of Points	% of Dataset
Naive	96	13
Injured	324	46
Sham	288	41

### Machine learning for brain injury classification: An introduction

Current home cage automated systems generate a large amount of behavioural data by continuous and non-invasive monitoring of mice that were subjected to a TBI. Uninjured animals are also monitored under the same experimental conditions as baseline data. Machine learning algorithms emerge as the natural choice for learning patterns from the recorded behavioural signals that would in turn be used to predict the presence of brain injury in a subject. Such models are typically trained on a training dataset and the model generalisability is evaluated on an independent test set that does not overlap with training data. Nevertheless, classical ML models and their deep learning counterparts involve different levels of data pre-processing and feature engineering steps. While deep neural networks are designed to capture salient features automatically from raw data, classical ML algorithms usually expect manually extracted features from raw signals as their input. Such features typically include statistical parameters such as the mean, standard deviation, range, intervals, etc,. However, deriving features manually could be tedious and the extracted parameters may not capture significant details inherent in the original signals. In contrast, deep neural networks, more specifically, convolutional layers work as remarkable feature extractors, eliminating the need of hand-crafted features. Briefly, a convolutional layer contains several randomly initialised kernels in the form a matrix (e.g. 3x3, 5x5 or 3x1) that are updated during the training cycle to capture various details from input data. The extracted features (also known as feature maps) are passed on to the next layers for a more abstract learning of the signals for classifying TBIs. Finally, these learned features are aggregated via a post-processing neural network (typically a set of fully-connected layers) to produce the output, which is the probability that the recorded behaviour data indicates the presence of a brain injury. In what follows, we present our approach in applying classical ML models and a novel deep neural network on our behavioural dataset for TBI detection.

#### SVM, decision tree and feedforward neural network

In order to prepare the data for training on our non-deep learning models, the average for each feature in a bin is calculated and then taken as a single data point. The resulting collection of data from binning forms the dataset, which is used to train our machine learning models. For a provision evaluation, we split our dataset into training, testing and validation sets, with ratios of 0.7, 0.15, 0.15, respectively. To ensure our data is internally consistent, all features in our dataset are standardised to the training dataset. Early analysis of the dataset revealed the presence of a heavy class imbalance against the naive class. We therefore proceeded to produce a second dataset where we perform oversampling to compensate for the heavily imbalanced dataset. We use Synthetic Minority Over-sampling Technique (SMOTE) as our chosen oversampling technique. SMOTE performs oversampling by taking a sample of a specific class, and using the vector between it and one of its neighbouring data points to generate a new synthetic data point. The addition of the new synthetic data for the infrequent class balances out the dataset, diminishing the frequency bias that the frequent classes have. On the regular, non-oversampled dataset, we instead adapt the loss function by assigning weights for each class, effectively giving the minority class a larger weight when learning.

#### Deep learning

In order to accomplish deep learning using sequential data, a separate dataset with sequential attributes was generated. This additional dataset is required because our deep learning model would take into consideration sequences and contextual information for classification, which a record style dataset would not be able to provide. Instead, data that better represents a series of data points, such as the time domain signals used for emotion classification in our related works, would be more appropriate to be used with our deep learning model. We assemble this information by using the complete information of each bin as a single data point, rather than compiling them into a single value. This results in a dataset where each data point houses all records of data within the bin. Therefore, each point of data possesses complete information about the fluctuations in distance travelled, temperature change, separation from other subjects and transitions, within the timeframe of its respective bin. This data however, still needs to be processed before being used to train our model. We pad each data point with zeros to ensure a consistent length of 48. We then split the data into training, testing and validation datasets for provisional evaluations. Standardisation is fitted to the training dataset, which is then used to standardise the testing and validation datasets accordingly. The oversampling technique SMOTE is not appropriate to be applied to this dataset and therefore we forego over-sampling this dataset. Class-weights are then calculated, which are used when training our deep learning model.

### Support vector machine

We implement the support vector machine (SVM) for the dataset using the SVM module from scikit-learn Python package. We use their implementation of support vector classification (SVC) which performs multi-class classification through the use of a one-vs-one scheme. Because SVMs are linear classification models, the one-vs-one scheme enables multi-class classification of SVMs by computing and combining binary classifications for each pair of classes. For our model we use the default C value of 1.0.

### Random forest classifier

Similar to our SVM implementation, we employ scikit-learn for our implementation of Random Forest. We use the random forest classifier method from the ensemble method with a max depth of 4.

### Feedforward neural network model architecture

Our implementation of a feedforward neural network model consists of a total of three layers, two hidden dense layers and an output dense layer. Two batch normalisation layers were added between the dense layers to include normalisation in the model architecture, resulting in a more stable model with reduced training times [32]. A softmax activation function is used for the output dense layer, which provides support for multi-class classification required. This results in an output of a probability distribution of the 3 classes in our dataset for each prediction that is made. For a given prediction, the class with the highest probability is then taken as the predicted class for that instance. The model was trained to minimise sparse categorical crossentropy loss for multi classification consisting of integer labels.

### Deep neural network model architecture

**Fig 3:**
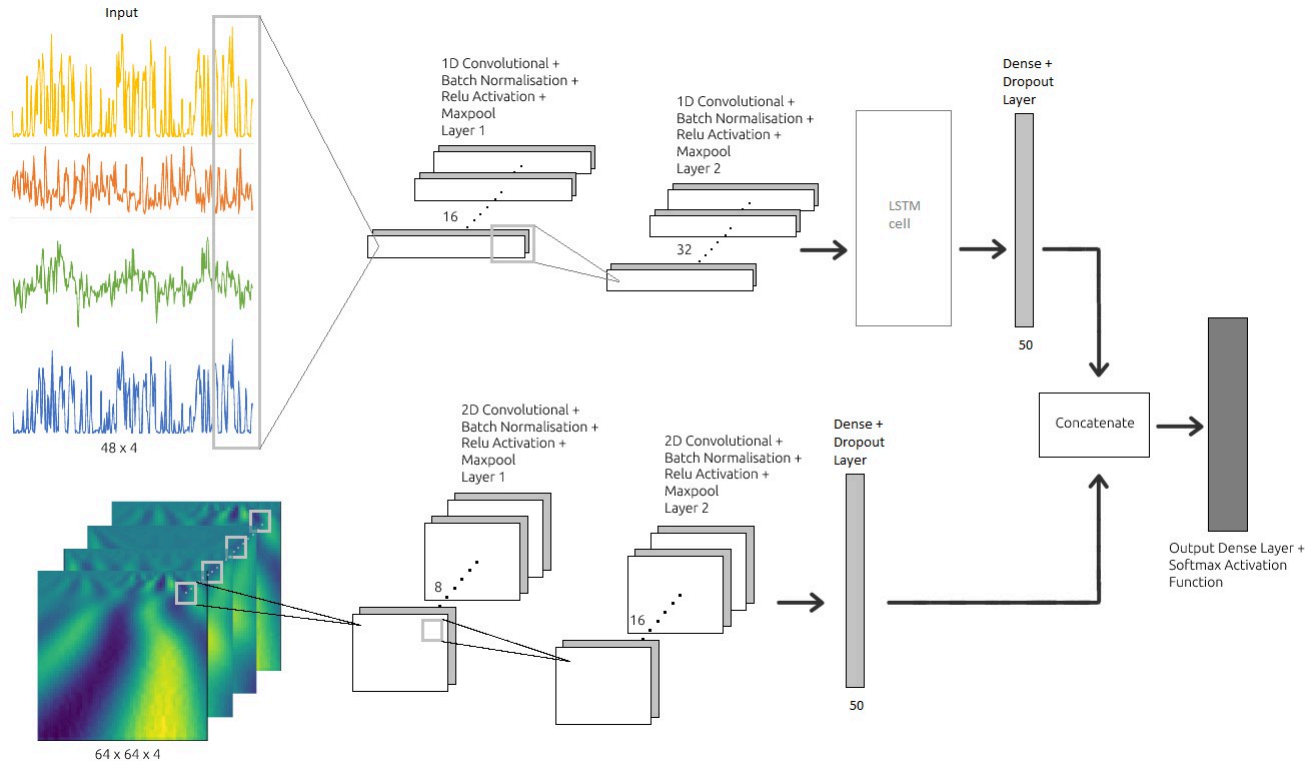
Architecture of the proposed deep neural network, with input shape and number of channels shown below the input, number of neurons below the dense layer. The features of time-dependent behavioural signals are captured via two convolutional-1D layers and a LSTM layer. CW transformed frequency domain signals are simultaneously processed by two convolutional-2D layers. The features extracted by both branches are then concatenated and sent through a final fully connected layer and a softmax function to return the probability of each class. The number of filters in each convolutional layer and units in LSTM and dense layers are tuned heuristically. The model is trained to minimize the sparse categorical crossentropy loss between the target class and the predicted class.

Our proposed deep neural network uses a combination of both the CNN and the RNN shown in Fig 3. The architecture takes precedent from the emotion classification model proposed by Kanjo et al. [23], in which their model processes time domain features such as PPG and GSR signals. This model also implements a similar structure to the ‘Y’ shaped emotion classification model that processes the raw RF signal by Khan et al. [25]. In our deep learning model, each instance from the produced deep learning dataset is fed first into the initial 1D Convolutional layer. A batch normalisation layer (*BN*) is added following the convolutional layer to make the training process faster and more stable [32], which is then followed by a ReLU (rectified linear unit) activation layer and a max pooling layer (*MaxPool*). This results in the following convolutional block:

$$\begin{array}{c} y=\sum_{i}\sum_{k}w_{i}x_{k}+b \end{array}$$ (1)

$$\begin{array}{c} s=BN(y) \end{array}$$ (2)

$$\begin{array}{c} h=ReLU(s) \end{array}$$ (3)

$$\begin{array}{c} f=MaxPool(h) \end{array}$$ (4)

where *w*_*i*_ indicates a trainable filter *i* and *x*_*k*_ indicates the input region that overlaps with the filter which is run through the input as a sliding window. This convolution block is repeated and then fed into the LSTM layer. A separate input is simultaneously fed into the model which also uses a similar convolution block. This second input takes in instances from the deep learning dataset that has undergone continuous wavelet transformation (CWT), which takes the form of an image and therefore uses a 2D convolutional layer instead. *L*_2_ regularisation is applied to the convolutional, LSTM and dense layers in order to counteract overfitting that may arise due to the small size of the dataset. The output 2D convolutional blocks are concatenated to the end of the output of the LSTM layer, which is then fed into a fully connected layer and a dropout layer. The final predicted label is once again produced by a softmax layer.

### Evaluation

In order to evaluate the performance of our models, we use leave-one-out cross validation (LOOCV) [33]. LOOCV is a type of cross validation, which is frequently used with machine learning models to determine their ability to generalise on unseen data as well as their overall performance. In LOOCV, each data point takes turns forming a singular test set. The remaining data is used as the training set to train the model, which then makes a prediction for the single test data. Therefore, the model is retrained for each instance in entire dataset. This results in an extremely high computation demand for large datasets, which is especially evident when used alongside models with high computational complexity. This also makes it relatively rare for LOOCV to be used with deep learning models. The benefit to using LOOCV however, would be that it essentially gives an unbiased estimate of the accuracy of the models [34]. Despite the complexity concerns, the relatively small size of the dataset used in this project allows for the practical use of LOOCV to evaluate our deep learning model, alongside the other machine learning models. For our implementation of LOOCV, standardisation and class weights are repeatedly calculated and applied to the dataset for each iteration.

## Results and discussion

**Fig 4:**
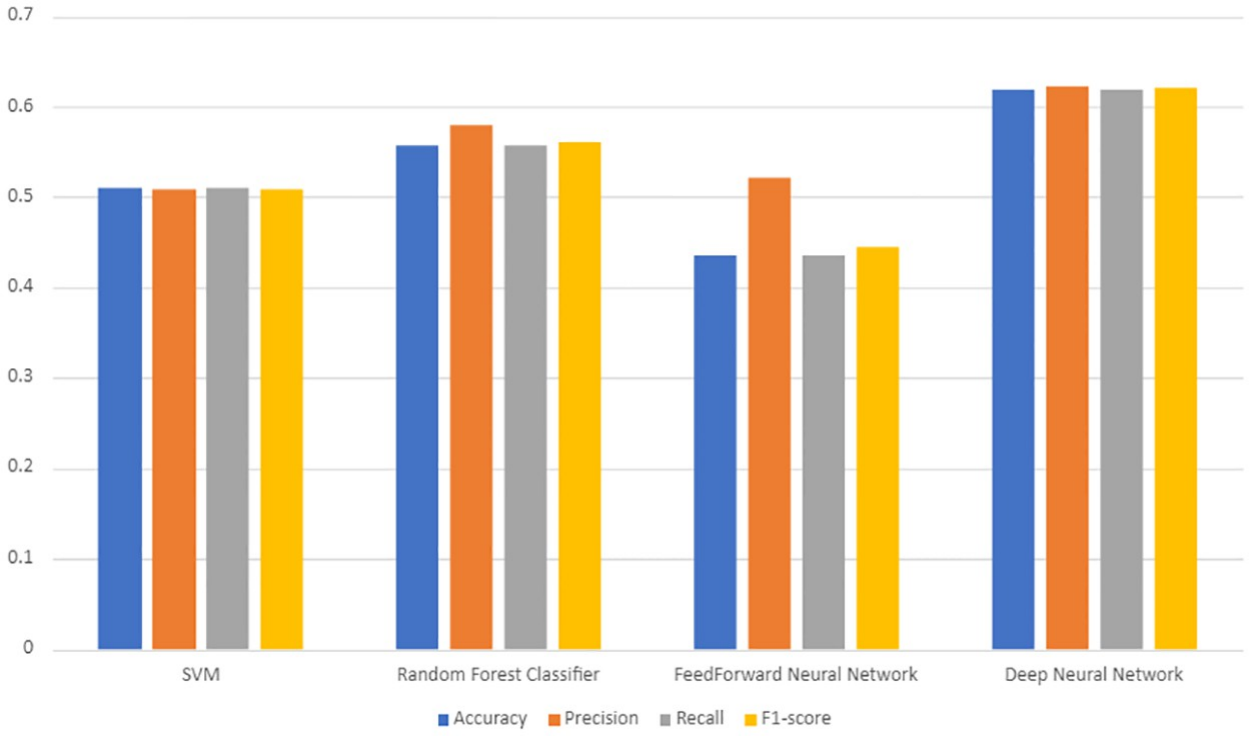
Performance metrics of the different machine learning techniques on LOOCV. Accuracy indicates the percentage of correct predictions. Precision is a measure of quality that indicates the percentage of relevant samples among selected samples. Recall is a measure of quantity that reflects the percentage of the relevant samples that were actually selected. F1-score is identified as a more sensible measure of accuracy of a model that is calculated using precision and recall. Higher the better for all metrics.

Through LOOCV, we obtain the performance for the models, on both the regular and sequential dataset, presented in Table 2 and Fig 4. LOOCV is not performed on the SMOTE dataset as it would not be a balanced comparison with the SMOTE-less deep learning model. In Fig 4 we present the accuracy, F1-score, precision and recall, calculated from the predictions made through LOOCV.

**Table 2:** Performances for machine learning models using leave-one-out cross validation.

	Accuracy (%)	Precision	Recall	F1-score
SVM	51.12	0.508	0.511	0.508
Random Forest Classifier	55.79	0.580	0.558	0.560
FeedForward Neural Network	43.50	0.522	0.435	0.445
Deep Neural Network	61.86	0.623	0.619	0.620

While the terms precision, recall and F1-score are originally defined for a binary classification task, these definitions can be extended for multi-class classification problems, like ours, by following a technique such as macro-averaging. Originally, precision is identified as a measure of quality that determines what fraction of selected items is relevant. It is formulated as;

$$\begin{array}{c} precision=\frac{true\;positives}{\left(true\;positives+false\;positives\right)} \end{array}$$ (5)

Recall is a measure of quantity that indicates what fraction of relevant items that was actually selected. It is postulated as;

$$\begin{array}{c} recall=\frac{true\;positives}{\left(true\;positives+false\;negatives\right)} \end{array}$$ (6)

F1-score determines the model’s accuracy on a dataset by combining precision and recall values. When precision and recall are very different, the F1-score tends towards the lower figure. F1-score is formulated as;

$$\begin{array}{c} F1\_score=\frac{2*precision*recall}{\left(precision+recall\right)} \end{array}$$ (7)

With an accuracy of 61.86%, results predictably identify our deep neural network as having the best performance amongst all the models. This is followed by the random forest classifier with the second best performance of 55.79%, and the SVM with an accuracy of 51.15%. The feedforward neural network performed the worst with an accuracy of 43.5%.

**Fig 5:**
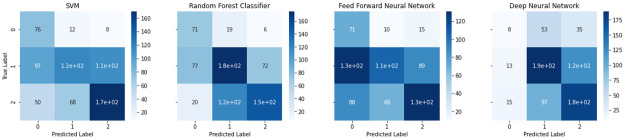
Confusion matrices obtained from predicted labels by performing Leave-one-out cross validation on the deep learning and machine learning models. Each confusion matrix reveals the performance of different ML algorithms on the same test set. It reflects the accuracy of an algorithm in predicting each class. The samples that fall in the main diagonal are correct predictions while off-diagonal instances are incorrect predictions. The proposed deep neural network can distinguish TBI and sham classes at an acceptable accuracy while naive class is difficult to capture.

**Fig 6:**
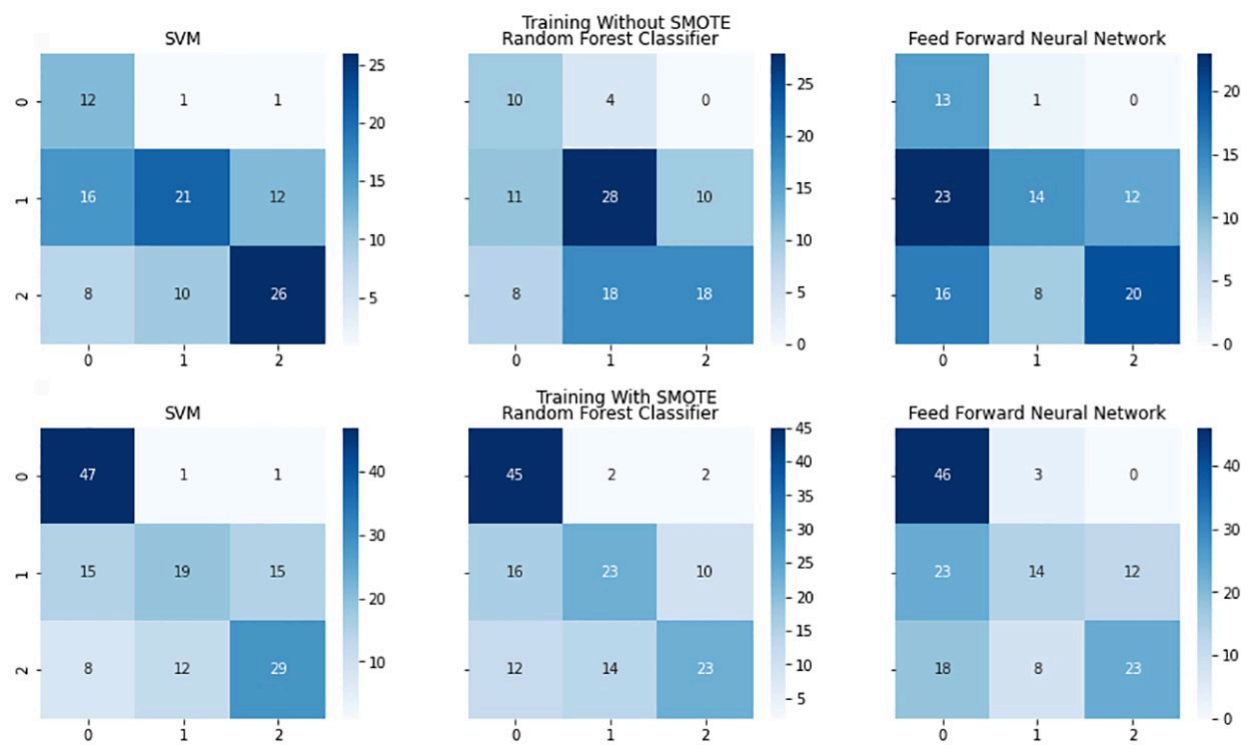
Confusion matrices of machine learning models trained on data oversampled by SMOTE and data trained with class weights given to the loss function. Because the naive class is oversampled using SMOTE, new data points are generated by interpolating between existing observations. As reflected in the confusion matrices, such an action results in ML models overfitting to the oversampled class, exhibiting an inflated accuracy on that class. The present oversampling approach has only a little effect on the performance on other two classes.

The confusion matrices obtained through LOOCV for all the machine learning models are depicted in Fig 5. To briefly assess the effect of SMOTE to the model performance, we also present the confusion matrices for the three non-deep learning models trained on SMOTE Fig 6.

The results of the models evaluated on LOOCV indicate our deep learning neural network as the preferred model to predict the presence of brain injury in the murine preclinical model. This is an expected result, because our deep learning architecture captures time dependency of raw data via convolutional and LSTM blocks. The data processed through CWT also presents the deep learning model with additional features not found in the raw data, providing the deep learning model additional information to learn from, further increasing its disparity from the other models. The much lower performance of the feedforward neural network when compared to its lower computation complexity counterparts is, however, unexpected. The relatively small size of the dataset, despite the extensive time based acquisition, could be an explanation for the lacking results of the neural network. With an increasing dataset size, the performance of classical machine learning algorithms such as the SVM and random forest classifier typically increase according to a power law before reaching a plateau [35]. In contrast to classical machine learning algorithms, the performance of neural networks has shown to increase logarithmically with the increase of training data [36]. Therefore, we can infer that classical machine learning algorithms such as the random forest classifier would require less training data to reach their optimal performance, and hence were able to achieve a considerable performance over the feed forward neural network with the limited amount of training data available. Their inexpensive demand for data however, is not without its disadvantages, because having more data beyond a certain point would not increase the performance of these techniques as they are constrained by the nature of the algorithms themselves. The constraints imposed by the dataset size in all classes result in the feedforward neural network not being able to generalise well, therefore justifying its poor performance. With more data, we can expect the feedforward neural network to outperform the random forest classifier, and also see an increase in performance from our deep neural network.

**Fig 7:**
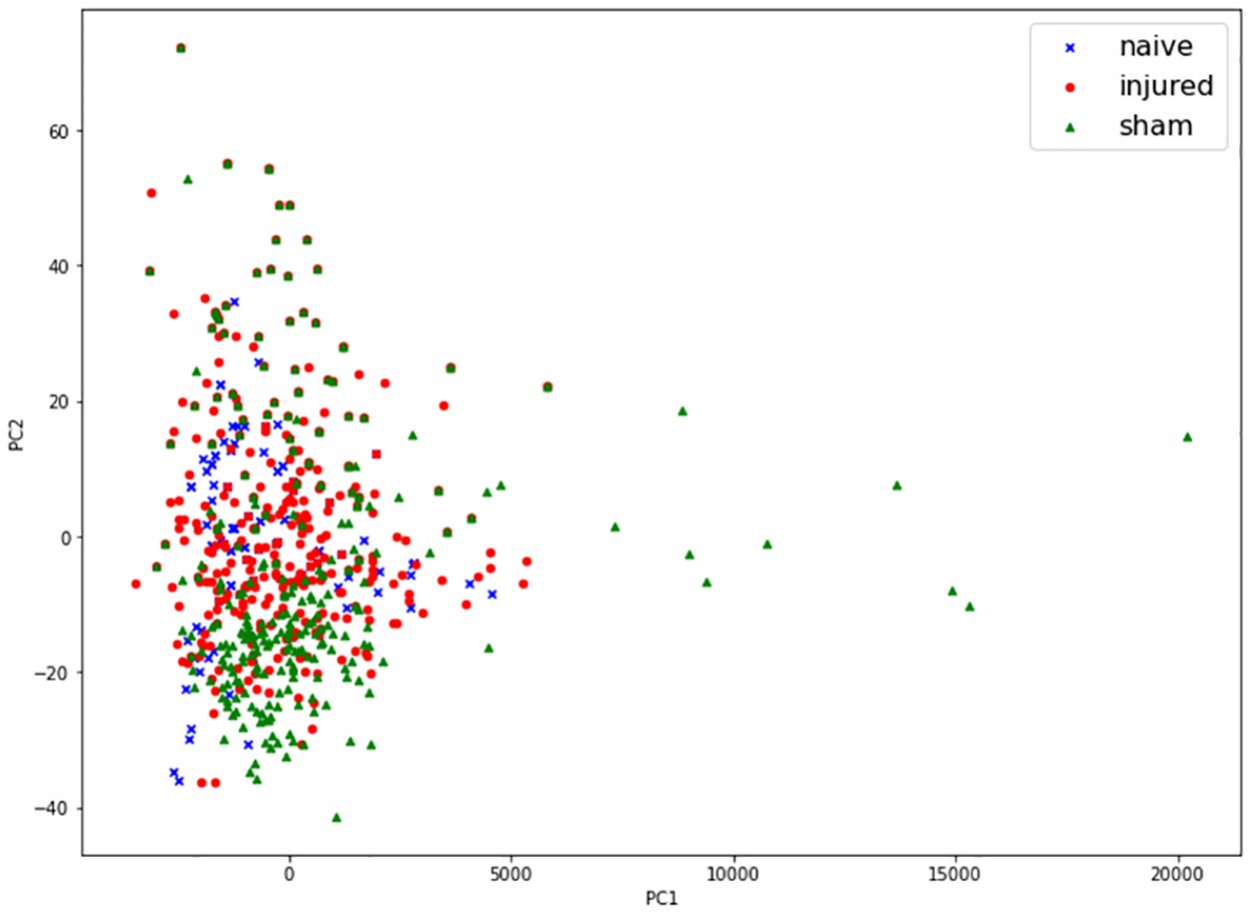
Principal component analysis plot of the dataset with two components. Two clusters, injured and sham have started forming although there is some overlap between the two groups. Naive data currently overlaps with other two major classes, however, more data may provide a better visual representation of the location of the naive cluster. Overall, it is not straightforward to distinguish the three experimental groups on the PCA map, indicating that the classification task is challenging.

A brief analysis of the data through the use of principal component analysis (PCA) shows latent information on how the classifiers can further benefit from more data (Fig 7) [37]. Through the use of PCA we can represent the dataset in the form of points in maps and therefore infer patterns of similarity between the observations [38, 39]. Aside from the prominent main cluster, smaller vague clusters nested within the main cluster can be found. The cluster for the sham class can be found between -10 to -30 on the y-axis and the cluster for the injured class right above that. Additionally, a much smaller cluster for the naive class, which is in the process of being formed, could be found above the injured cluster. With more data, specifically the naive data, it is possible that the third cluster would be formed, entailing a better accuracy for the label and hence a better overall performance of the model. Whilst all models should be able to benefit from additional data, the neural networks should benefit the most from them. We can also infer the reason for the higher accuracy on sham and injured classes from the PCA analysis, which can be observed in the confusion matrices Fig 5. Most data points overlap with each other or are in the same cluster. However, sham and injured classes have several outliers, which would make them much easier to distinguish from the other classes. Points on the naive class; however, mostly lie in the main cluster, where naive data rightfully should not contain much variability. This results in the naive class being the hardest to differentiate from, causing them to be the most difficult class to predict.

This imbalance in the dataset and lack of variation in the naive groups could be offset by our use of the oversampling technique SMOTE. Our results from training the machine learning algorithms with the oversampled dataset shows a much better performance, especially on the naive class. We do not however use the SMOTE oversampled dataset to train and evaluate our deep learning model with LOOCV due to its tendency to overfit and result in overly-optimistic estimate on cross-validation [40]. This is also a factor in addition to the lack of a SMOTE oversampled sequential dataset. Undersampling would be a much more accurate technique to deal with the class imbalance if data were not limited [41].

## Conclusion

In this study, we applied both classical machine learning techniques and a novel deep neural network to predict the presence of TBI in mice using behavioural data acquired from an automated home cage recording system. The models learned to classify three injury states; injured (TBI), in which the mice have been subjected to a controlled cortical injury; sham, where the mice have undergone sham surgical intervention without brain injury; and naive, which are not subjected to any injury. The proposed deep learning architecture benefits from the time dependency of raw data processed via LSTM and convolutional-1D layers and the spatial relationships present in the frequency-domain CWT images processed via convolutional-2D layers.

We further note that, while a data oversampling technique such as SMOTE may be employed to resolve the class imbalance issue in naive class, this may not reflect the true error in predicting the naive class and display an inflated accuracy due to overfitting to that class. Leave-one-out cross validation was identified as an unbiased evaluation scheme for all algorithms. Our model outperformed alternative ML models such as SVM and random forest by at least 6% in classifying three brain injury states. The capability of the proposed model to preserve temporal and spatial features of raw data, as opposed to classical ML models that break such relationships, could be identified as the main reason for its improved performance. Predicting the presence of TBI using behavioural data is recognised as a challenging task and all ML models are expected to display an improved performance as more data becomes available. Finally, as future work, we plan to predict the degree of severity of brain injuries (e.g. mild vs moderate vs severe) using behavioural data, and incorporate recovery in the prediction task.

## Supporting information

S1 Fig Behavioural and histological evidence of the severity of the CCI model used in this study. A modified Neurological Severity Score (mNSS1) was used to assess the motor ability, alertness and balance of each animal on day 1, 3, and 5 post intervention and then once a week, until the end of the experiment. A representative histological representation of the brains for the different experimental groups is included—showing the degree of injury in the brain of the CCI-Injured animals.
(PNG)

S1 File The HCA data collected in this study is provided as supporting data. (ZIP)
