# Supplementary figures and images for: Deep learning for behaviour classification in a preclinical brain injury model
Source: PLoS One. 2022 Jun 15;17(6):e0268962. doi: 10.1371/journal.pone.0268962 (PMC9200342; doi:10.1371/journal.pone.0268962)

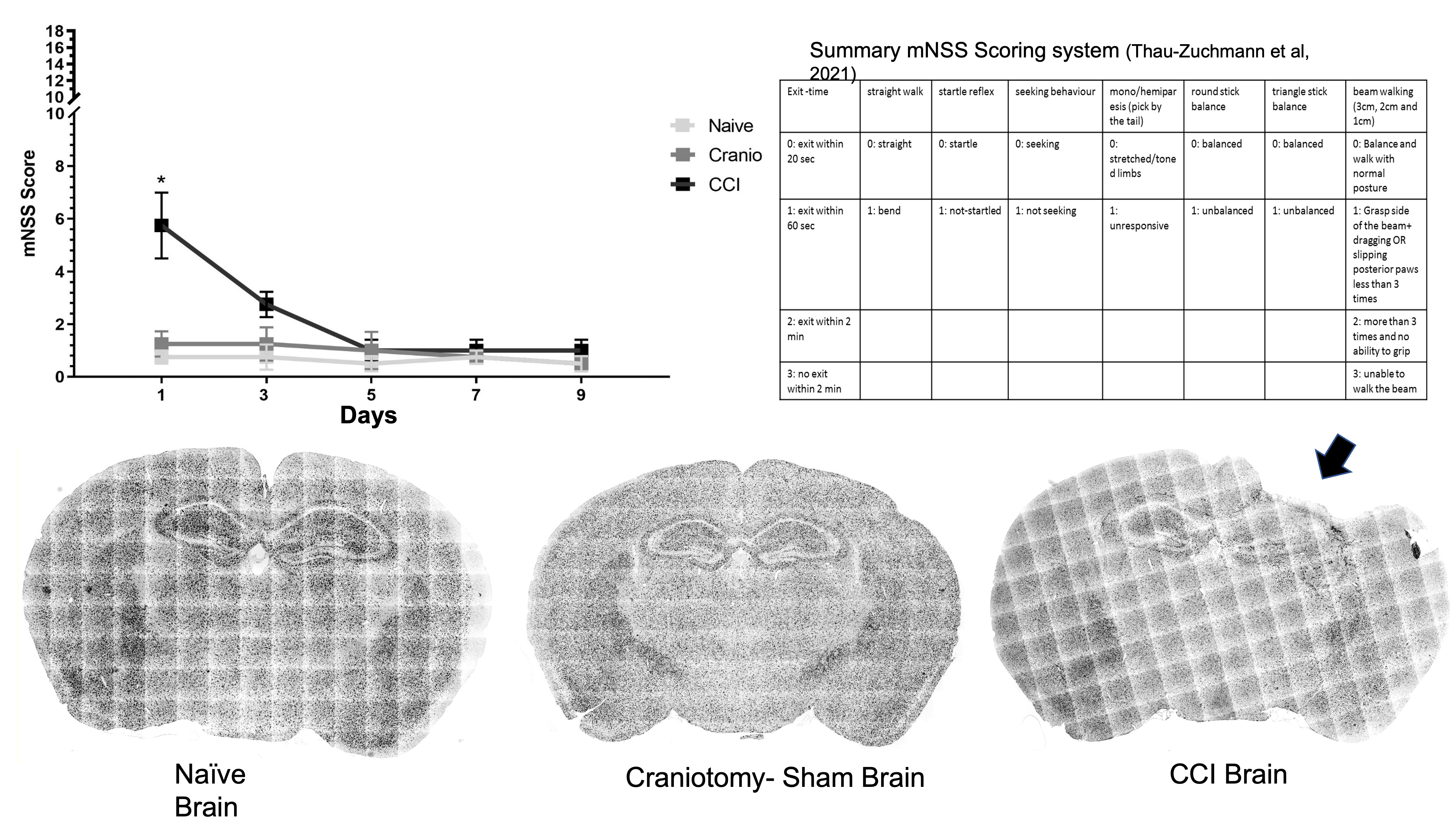

Supplement: S1 Fig — A modified Neurological Severity Score (mNSS1) was used to assess the motor ability, alertness and balance of each animal on day 1, 3, and 5 post intervention and then once a week, until the end of the experiment. A representative histological representation of the brains for the different experimental groups is included—showing the degree of injury in the brain of the CCI-Injured animals. (PNG) [file pone.0268962.s001.png]
